# Elucidating structure–property relationships of guar gum biomolecules: insights from M -polynomial and QSPR modeling

**DOI:** 10.3389/fchem.2024.1410876

**Published:** 2024-07-09

**Authors:** Waheed Khalid, Shamaila Yousaf

**Affiliations:** Department of Mathematics, University of Gujrat, Gujrat, Pakistan

**Keywords:** chemical graph theory, QSPR, physicochemical properties, M-polynomials, topological indices, guar gum

## Abstract

This study investigates the quantitative structure–property relationship (QSPR) modeling of guar gum biomolecules, focusing on their structural parameters. Guar gum, a polysaccharide with diverse industrial applications, exhibits various properties such as viscosity, solubility, and emulsifying ability, which are influenced by its molecular structure. In this research, M -polynomial and associated topological indices are employed as structural descriptors to represent the molecular structure of guar gum. The M -polynomial and associated topological indices capture important structural features, including size, shape, branching, and connectivity. By correlating these descriptors with experimental data on guar gum properties, predictive models are developed using regression analysis techniques. The analysis revealed a strong correlation between the boiling point and molecular weight and all the considered topological descriptors. The resulting models offer insights into the relationship between guar gum structure and its properties, facilitating the optimization of guar gum production and application in various industries. This study demonstrates the utility of M -polynomial and QSPR modeling in elucidating structure–property relationships of complex biomolecules like guar gum, contributing to the advancement of biomaterial science and industrial applications.

## 1 Introduction

Guar gum, an innovative agrochemical, is derived from the endosperm of cluster beans. The species *Cyamopsis tetragonoloba*, belonging to the Leguminosae family, produces the seeds used in guar gum production, and these seeds are resistant to drought (Prem et al., 2005). The concept of transdomestication was introduced by Hymowitz, although the exact origins of this practice are still a matter of dispute. Further information on the subject can be found in Whistler (1948) and BeMiller (2009). Guar gum has recently piqued the curiosity of several experts for numerous reasons. Guar gum powder serves as a thickener, stabilizer, and health management tool in various industries. It consists of galactomannan polysaccharides and can be derived from different sources, including algae, plants, microorganisms, and animals (Hovgaard and Brondsted, 1996). These polysaccharides are characterized by their stability, non-toxicity, hydrophilicity, and biodegradability. Its unique properties, such as high viscosity, solubility, and stability, make it a versatile ingredient with a wide range of applications in industries like food, pharmaceuticals, cosmetics, and oil drilling. Additionally, guar gum is known for its biocompatibility and non-toxic nature, making it suitable for various biomedical and pharmaceutical applications. By studying guar gum, researchers can explore its potential uses, optimize its properties, and develop innovative products with improved performance and functionality. Overall, the selection of guar gum for research offers opportunities to advance knowledge in various fields and contribute to the development of sustainable and high-performing materials and products.

Various derivatives of guar gum exist, such as carboxymethyl guar HPG , hydroxymethyl guar CMG , hydroxypropylethyl guar, ammonium hydroxylpropyl trimethyl chloride guar, acryloyloxy guar, methacryloyl guar, guar gum esters, and carboxymethyl hydroxypropyl guar CMHPG . The computation of guar gum and its derivatives employs molecular graphs, as detailed in Shanmukha et al. (2022). The most widely used derivatives are carboxymethyl guar (CMG) , hydroxypropyl guar (HPG) , and carboxymethyl hydroxypropyl guar (CMHPG) , which are discussed in this study with respect to topological indices.

Chemical graph theory is an interdisciplinary field that links chemistry and mathematics. Graph modeling, originating from early chemical experiments, is a crucial aspect of theoretical chemistry. The subfield of cheminformatics analyzes quantitative structure–activity relationships (QSAR) and quantitative structure–property relationships (QSPR) to predict the biological activity and characteristics of guar gum and its derivatives. Utilizing topological indices and physico-chemical substances, it is possible to infer the pharmacological activity of these compounds without conducting experiments. Noteworthy, studies related to topological indices and physico-chemical substances include Arockiaraj et al. (2023a); Arockiaraj et al. (2023b); and Arockiaraj et al. (2023c). Recently, algebraic polynomials, such as the Hosoya polynomial (Consonni and Todeschini, 2010a) and M -polynomials (Deutsch and Klavar, 2014), have gained prominence in chemistry for determining distance-based topological indices and degree-based topological indices, respectively. The M -polynomial yields closed forms for various degree-based indices, while the Hosoya polynomial focuses on distance-based indices. The M -polynomial, often denoted as M(x,y) , is a polynomial used in the study of chemical graph theory, particularly in the enumeration of certain chemical structures known as molecular graphs. The M -polynomial encodes information about the molecular graph’s topology, such as its number of vertices, edges, and other structural properties. It has applications in the enumeration of molecular isomers, the prediction of molecular properties, and the study of chemical reactions. Significant knowledge regarding degree-based graph invariants can be found in the M -polynomial literature, including Munir et al. (2016a); Munir et al. (2016b); Munir et al. (2016c); Munir et al. (2016d); and Ajmal et al. (2017). There is a wealth of knowledge regarding degree-based graph invariants in the M -polynomial.

The M -polynomial of graph Γ is defined as

$$M\left(\Gamma ;x,y\right)=\underset{s\leq t}{\sum }m_{st}\left(\Gamma \right)x^{s}y^{t},$$

where *m*_*st*_(Γ) is the number of edges *νυ* ∈ *E*(Γ) such that {*d*_*ν*_, *d*_*υ*_} = {*s*, *t*}.

Table 1 contains some degree-based TIs and the M -polynomial for the graph Γ: $D_{x}=x\frac{\partial \left(g\left(x,y\right)\right)}{\partial \left(x\right)}$ , $D_{y}=y\frac{\partial \left(g\left(x,y\right)\right)}{\partial \left(y\right)}$ , $S_{x}=\int_{0}^{x}\frac{g\left(t,y\right)}{t}dt$ , $S_{y}=\int_{0}^{y}\frac{g\left(x,t\right)}{t}dt,I\left(g\left(x,y\right)\right)=\left(g\left(x,x\right)\right)$ , and $Q_{\alpha }\left(g\left(x,y\right)\right)=x^{\alpha }\left(g\left(x,y\right)\right)$ .

**TABLE 1:** M -polynomials are used to derive several degree-based topological indices.

*TopologicalIndex*	DerivationfromM(Γ;x,y)
*M*_1_	$\left(D_{x}+D_{y}\right)\left(M\left(\Gamma ;x,y\right)\right){|}_{x=y=1}$
*M*_2_	$\left(D_{x}D_{y}\right)\left(M\left(\Gamma ;x,y\right)\right){|}_{x=y=1}$
^*m*^ *M*_2_	$\left(S_{x}S_{y}\right)\left(M\left(\Gamma ;x,y\right)\right){|}_{x=y=1}$
*R*_*α*_	$\left(D_{x}^{\alpha }D_{y}^{\alpha }\right)\left(M\left(\Gamma ;x,y\right)\right){|}_{x=y=1}$
*RR*_*α*_	$\left(S_{x}^{\alpha }S_{y}^{\alpha }\right)\left(M\left(\Gamma ;x,y\right)\right){|}_{x=y=1}$
*SDD*	$\left(D_{x}S_{y}+S_{x}D_{y}\right)\left(M\left(\Gamma ;x,y\right)\right){|}_{x=y=1}$
*H*	$2\left(S_{x}I\right)\left(M\left(\Gamma ;x,y\right)\right){|}_{x=1}$
*I*	$\left(S_{x}ID_{x}D_{y}\right)\left(M\left(\Gamma ;x,y\right)\right){|}_{x=1}$
*AZI*	$\left(S_{x}^{3}Q_{-2}ID_{x}^{3}D_{y}^{3}\right)\left(M\left(\Gamma ;x,y\right)\right){|}_{x=1}$

The topological index, frequently known as the connectedness index, was introduced in 1947 as a result of Weiner’s research (Consonni and Todeschini, 2010b). The earliest and most extensively researched topological index was the Wiener index [for further information, see (Wiener, 1947; Gutman et al., 1986)]. One of the earliest topological indices, the Randi $\overset{´}{c}$ index (Randic, 1975), was first introduced by Milan Randi $\overset{´}{c}$ in 1975 and is represented by the symbol $R_{-\frac{1}{2}}\left(\Gamma \right)$ . Its definition is as follows:

$$R_{-\frac{1}{2}}\left(\Gamma \right)=\underset{\upsilon \nu \in E\left(\Gamma \right)}{\sum }\frac{1}{\sqrt{d_{\upsilon }d_{\nu }}}.$$ (1)

In 1998, Bollobs and Erds (1998) and Amic et al. (1998) independently proposed the general Randi $\overset{´}{c}$ index, which has been extensively studied for its numerous mathematical properties (Caporossi et al., 2003; Hu et al., 2005). For a detailed survey, refer to Li et al. (2006). The general Randi $\overset{´}{c}$ index is defined as

$$R_{\alpha }\left(\Gamma \right)=\underset{\upsilon \nu \in E\left(\Gamma \right)}{\sum }\frac{1}{{\left(d_{\upsilon }d_{\nu }\right)}^{\alpha }},$$ (2)

and $RR_{\alpha }\left(\Gamma \right)=\sum_{\upsilon \nu \in E\left(\Gamma \right)}{\left(d_{\upsilon }d_{\nu }\right)}^{\alpha }$ is the definition of the inverse Randi $\overset{´}{c}$ index.

Many papers and books, such as Kier and Hall (1976) and Kier and Hall (1986), have been produced on this topological index. For drug design, the Randi $\overset{´}{c}$ index was recognized. The first and second Zagreb indices were introduced by Gutman and Trinajsti $\overset{´}{c}$ . They are denoted as follows: *M*_1_(Γ) = *∑*_*υν*∈*E*(Γ)_(*d*_*υ*_ + *d*_*ν*_) and *M*_2_(Γ) = *∑*_*υν*∈*E*(Γ)_(*d*_*υ*_ *d*_*ν*_), respectively. The reader is referred to Nikolic et al. (2003); Das and Gutman (2004); Gutman and Das (2004); Vukicevic and Graovac (2004); and Trinajstic et al. (2010) for further information on these indices. Among several modifications of Zagreb indices, one is the second modified Zagreb index. According to Milicevic et al. (2004), the second modified Zagreb index for a simple connected graph Γ is defined as

M2mΓ=∑υν∈EΓ1dυdν (3)

The symmetric division index (SDD) is particularly useful in determining the total surface area for polychlorobiphenyls (Gupta et al., 2016) based on the discrete Adriatic indices. The symmetric division index of a connected graph *G* is defined as

$$SDD\left(\Gamma \right)=\underset{\upsilon \nu \in E\left(\Gamma \right)}{\sum }\left\{\frac{\min\left(d_{\upsilon },d_{\nu }\right)}{\max\left(d_{\upsilon },d_{\nu }\right)}+\frac{\max\left(d_{\upsilon },d_{\nu }\right)}{\min\left(d_{\upsilon },d_{\nu }\right)}\right\}.$$ (4)

The harmonic index is an additional Randi $\overset{´}{c}$ index variation that is described as

$$H\left(\Gamma \right)=\underset{\upsilon \nu \in E\left(\Gamma \right)}{\sum }\frac{2}{d_{\upsilon }+d_{\nu }}.$$ (5)

There is a relationship between graph eigenvalues and the harmonic index (Favaron et al., 1993). Using MathChem, the elegant structure of extremal graphs is used to generate the inverse sum index (Balaban, 1982), a significant predictor of the octane isomer total surface area.

$$I\left(\Gamma \right)=\underset{\upsilon \nu \in E\left(\Gamma \right)}{\sum }\frac{d_{\upsilon }d_{\nu }}{d_{\upsilon }+d_{\nu }}.$$ (6)

Furtula et al. (2010) is credited for the augmented Zagreb index *AZI*, which is characterized as

$$AZI\left(\Gamma \right)=\underset{\upsilon \nu \in E\left(\Gamma \right)}{\sum }{\left\{\frac{d_{\upsilon }d_{\nu }}{d_{\upsilon }+d_{\nu }-2}\right\}}^{3}.$$ (7)

Graph invariant *AZI* has higher prediction power than the atom-bond connectivity index (Furtula et al., 2010) and is a useful predictive measure for analyzing the heat of formation in octanes and heptanes (for more detail, see (Estrada et al., 1998; Das, 2010)). A few well-known degree-based topological indices (which are defined in Eqs 1–7) with M -polynomials (Deutsch and Klavar, 2014) are related in the following Table 1.

## 2 Methodology

**FIGURE 1:**
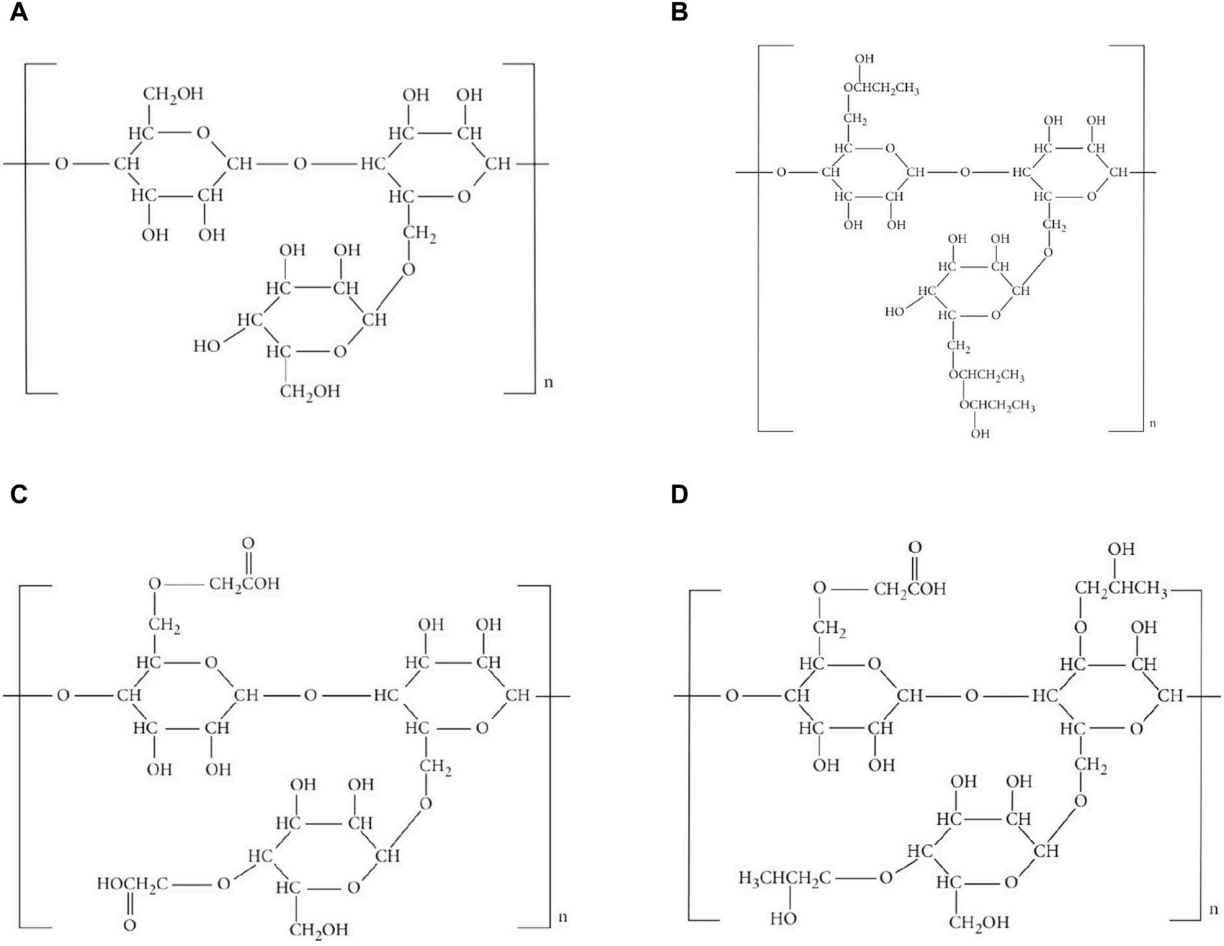
Molecular structure. **(A)** guar gum, **(B)** HPG, **(C)** CMG, and **(D)** CMHPG.

**FIGURE 2:**
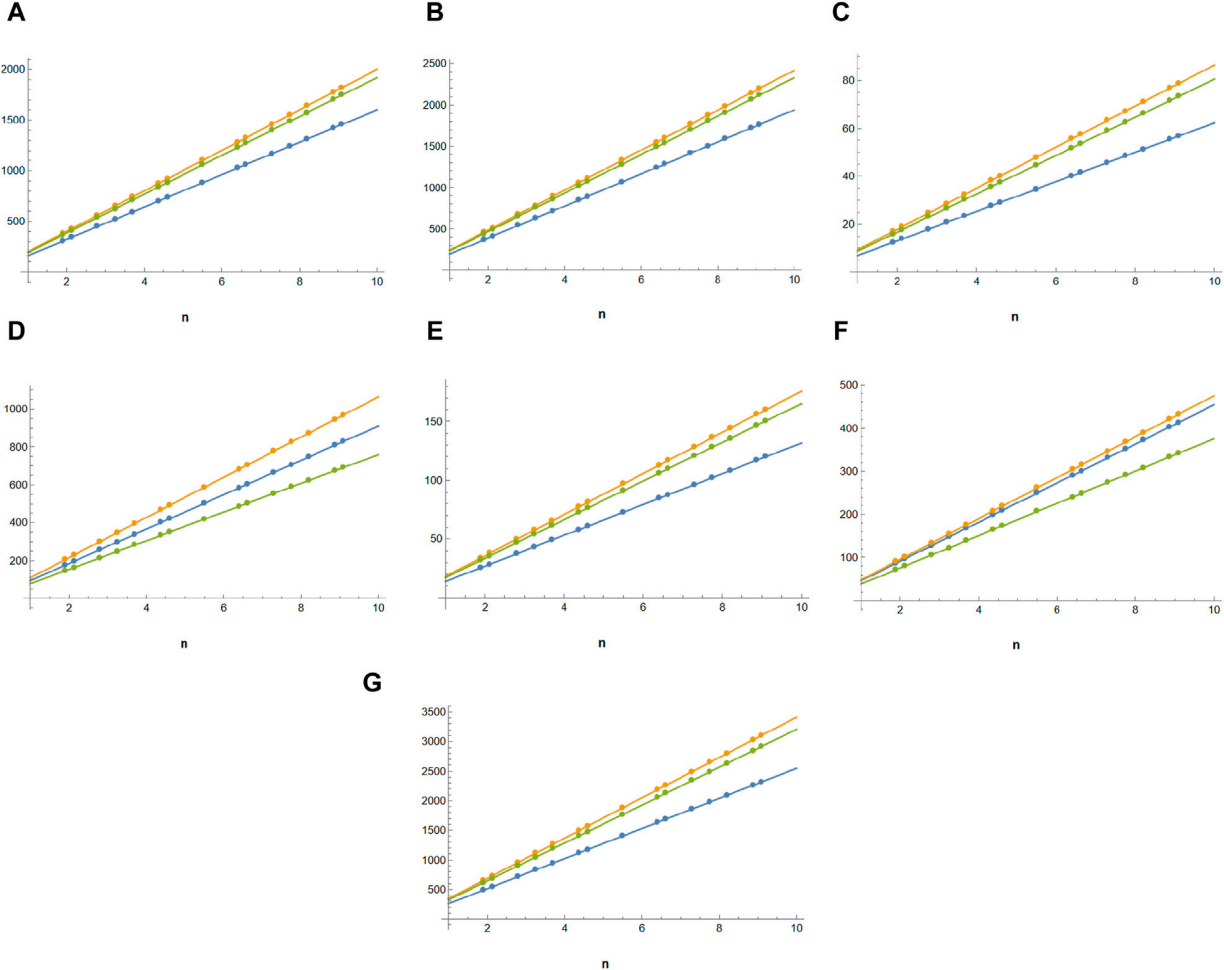
Comparison of topological indices of guar gum in blue, HPG and CMG in yellow, and CMHPG in green, respectively. **(A)** *M*_*1*_, **(B)** *M*_*2*_, **(C)** ^*m*^ *M*_*2*_, **(D)** *SDD*, **(E)** *H*, **(F)** *I*, and **(G)** *AZI*.

**FIGURE 3:**
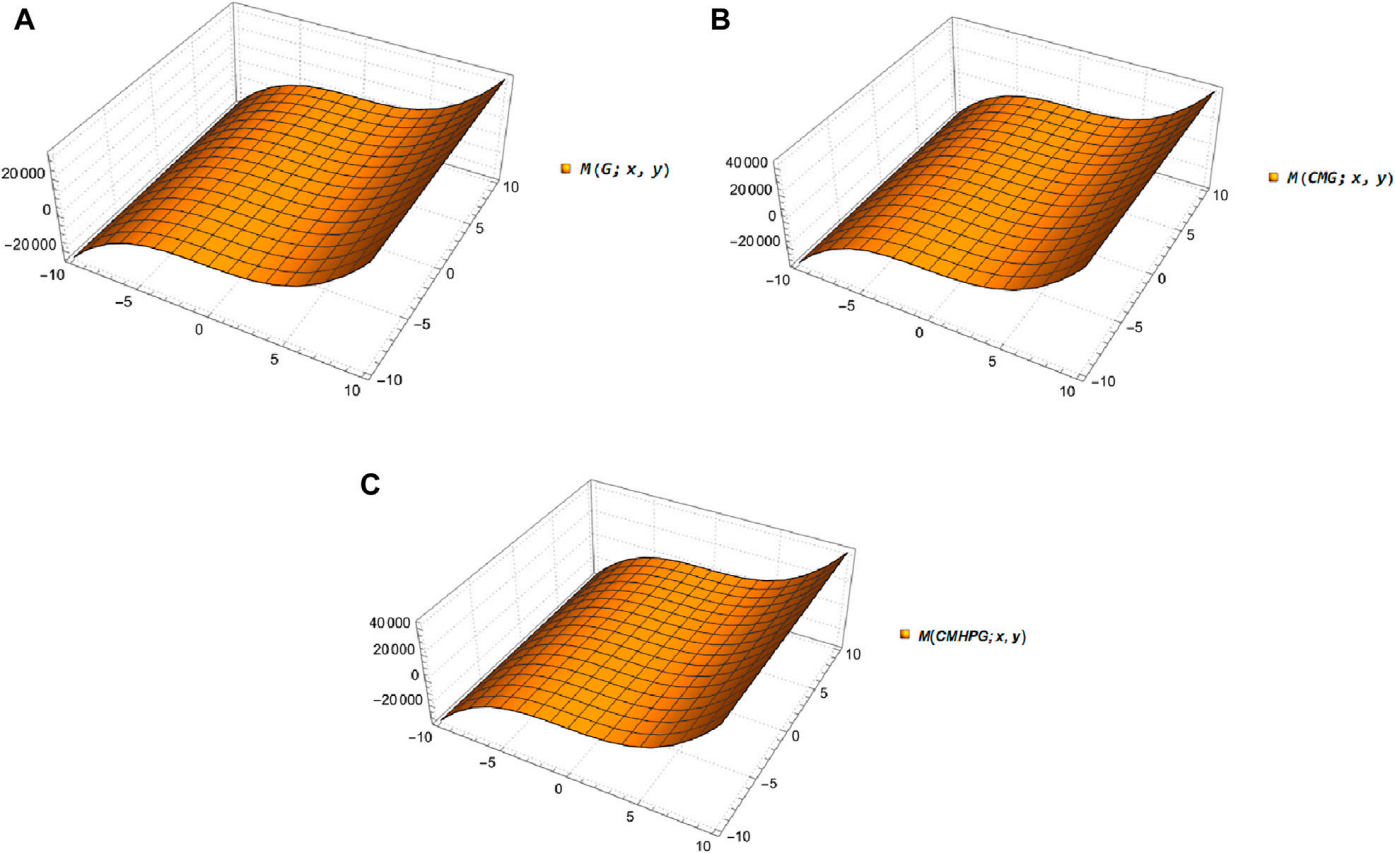
3D plots of M -polynomials. **(A)** Guar gum, **(B)** CMG, **(C)** CMHPG.

Molecular graphs and vertex and edge partitions are used to modify the molecular structure of guar gum and its chemical derivatives (Figure 1). Topological indices are derived using M -polynomial. Graphical comparisons of the aforementioned defined indices are made using vertex partition, edge partition, and combinatorial computing. The graphical representation of the outcomes and the comparative study of the findings are performed via 2D plotting in Figure 2 and 3D plotting in Figure 3 are shown by utilizing Mathematica software. In this study, physio-chemical properties of the selected guar gum and its derivatives were obtained from ChemSpider, providing a comprehensive dataset for analysis. Several topological indices were calculated using M -polynomials, extracting key molecular information relevant to biological activities. Subsequently, these indices were utilized in quantitative structure–property relationship (QSPR) analysis, employing SPSS software. The process involves constructing linear, quadratic, and logarithmic models to establish correlations between the calculated topological indices and the properties of selected guar gum and its derivatives. This meticulous methodology aims to uncover patterns and relationships within the molecular structures, contributing to a deeper understanding of the properties of selected guar gum and its derivatives.

## 3 Main results and discussions

This section outlines our primary analytical findings and subdivides the material into three sections: guar gum, hydroxypropyl guar, and carboxymethyl guar. The M -polynomials and their associated topological indices are derived for the chemical structures of guar gum, which are useful in the QSPR study. Guar gum is a polysaccharide used in various industries including food, pharmaceuticals, and cosmetics. QSPR studies based on M -polynomials and topological indices can help in understanding how its structural features relate to its properties such as viscosity, solubility, and emulsifying ability. By establishing quantitative relationships between structure and properties, researchers can optimize the production and application of guar gum for various industrial purposes.

### 3.1 Guar gum

**FIGURE 4:**
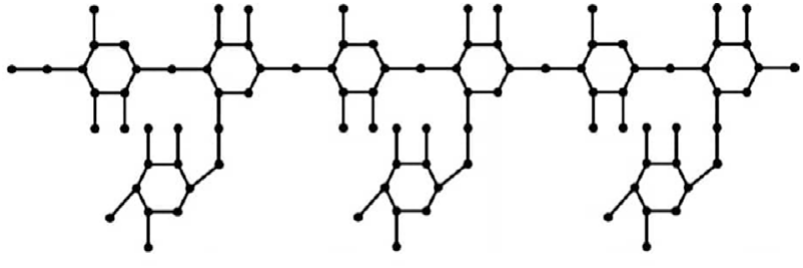
Guar gum molecular graph for *n* = 3.

This section uses various topological indices to analyze the molecular graph of guar gum. Figure 4 shows the vertex and edge partitioning of guar gum.

$$E_{\left\{i_{0},i_{1}\right\}}=\left\{e=\upsilon \nu \in E\left(\Gamma \left[m,n\right]\right):d_{\upsilon }=i_{0},d_{\nu }=i_{1}\right\},$$

such that |*E*_{1,2}_| = 1, |*E*_{1,3}_| = 7*n* + 1, |*E*_{2,2}_| = 2*n*, |*E*_{2,3}_| = 14*n* − 1, and |*E*_{3,3}_| = 9*n*.

Theorem 3.2 *Consider a molecular graph* Γ *for guar gum. Then,*

$$M\left(\Gamma ;x,y\right)=xy^{2}+\left(7n+1\right)xy^{3}+2nx^{2}y^{2}+\left(14n-1\right)x^{2}y^{3}+9nx^{3}y^{3}.$$

Proof. Using the M -polynomial of Γ, we obtain the following equation:

$$\begin{array}{ll} M\left(\Gamma ;x,y\right) & =\underset{i_{0}\leq i_{1}}{\sum }m_{i_{0}i_{1}}\left(\Gamma \right)x^{i_{0}}y^{i_{1}}.\\ & =\underset{1\leq 2}{\sum }m_{12}\left(\Gamma \right)x^{1}y^{2}+\underset{1\leq 3}{\sum }m_{13}\left(\Gamma \right)x^{1}y^{3}+\underset{2\leq 2}{\sum }m_{22}\left(\Gamma \right)x^{2}y^{2}+\underset{2\leq 3}{\sum }m_{22}\left(\Gamma \right)x^{2}y^{3}\\ & \;+\underset{3\leq 3}{\sum }m_{33}\left(\Gamma \right)x^{3}y^{3}.\\ & =\underset{uv\in E_{\left\{1,2\right\}}}{\sum }m_{12}\left(\Gamma \right)x^{1}y^{2}+\underset{uv\in E_{\left\{1,3\right\}}}{\sum }m_{13}\left(\Gamma \right)x^{1}y^{3}+\underset{uv\in E_{\left\{2,2\right\}}}{\sum }m_{22}\left(\Gamma \right)x^{2}y^{2}\\ & \;+\underset{uv\in E_{\left\{2,3\right\}}}{\sum }m_{23}\left(\Gamma \right)x^{2}y^{3}+\underset{uv\in E_{\left\{3,3\right\}}}{\sum }m_{33}\left(\Gamma \right)x^{3}y^{3}.\\ & =|E_{\left\{1,2\right\}}x^{1}y^{2}+|E_{\left\{1,3\right\}}|x^{1}y^{3}+|E_{\left\{2,2\right\}}|x^{2}y^{2}+|E_{\left\{2,3\right\}}|x^{2}y^{3}+|E_{\left\{3,3\right\}}|x^{3}y^{3}.\\ & =xy^{2}+\left(7n+1\right)xy^{3}+2nx^{2}y^{2}+\left(14n-1\right)x^{2}y^{3}+9nx^{3}y^{3}. \end{array}$$

Proposition 3.3 *Consider a molecular graph* Γ *for guar gum. Then,*

$$\begin{array}{ll} 1.M_{1}\left(\Gamma \right) & =160n+2.\\ 2.M_{2}\left(\Gamma \right) & =194n-1.\\ 3.^{m}M_{2}\left(\Gamma \right) & =6.167n+0.67.\\ 4.R_{\alpha }\left(\Gamma \right) & =2^{\alpha }+3^{\alpha }\left(7n+1\right)+2^{2\alpha +1}n+2^{\alpha }3^{\alpha }\left(14n-1\right)+3^{2\alpha +2}n.\\ 5.RR_{\alpha }\left(\Gamma \right) & =\frac{1}{2^{\alpha }}+\frac{\left(7n+1\right)}{3^{\alpha }}+\frac{n}{2^{2\alpha -1}}+\frac{\left(14n-1\right)}{2^{\alpha }3^{\alpha }}+\frac{n}{3^{2\alpha -2}}.\\ 6.SDD\left(\Gamma \right) & =75.67n+2.5. \end{array}$$

Proof: From the edge partitioning of guar gum and using the definition M -polynomial of Γ, we obtain the following equations (Eqs 8–16):

$$g\left(x,y\right)=M\left(\Gamma ;x,y\right)=xy^{2}+\left(7n+1\right)xy^{3}+2nx^{2}y^{2}+\left(14n-1\right)x^{2}y^{3}+9nx^{3}y^{3},$$

$$D_{x}\left(g\left(x,y\right)\right)=xy^{2}+\left(7n+1\right)xy^{3}+4nx^{2}y^{2}+2\left(14n-1\right)x^{2}y^{3}+27nx^{3}y^{3},$$ (8)

$$D_{y}\left(g\left(x,y\right)\right)=xy^{2}+3\left(7n+1\right)xy^{3}+4nx^{2}y^{2}+3\left(14n-1\right)x^{2}y^{3}+27nx^{3}y^{3},$$ (9)

$$D_{x}D_{y}\left(g\left(x,y\right)\right)=xy^{2}+3\left(7n+1\right)xy^{3}+8nx^{2}y^{2}+6\left(14n-1\right)x^{2}y^{3}+81nx^{3}y^{3},$$ (10)

$$S_{x}\left(g\left(x,y\right)\right)=xy^{2}+\left(7n+1\right)xy^{3}+nx^{2}y^{2}+\frac{1}{2}\left(14n-1\right)x^{2}y^{3}+\frac{9}{3}nx^{3}y^{3},$$ (11)

$$S_{y}S_{x}\left(g\left(x,y\right)\right)=\frac{1}{2}xy^{2}+\frac{1}{3}\left(7n+1\right)xy^{3}+\frac{1}{2}nx^{2}y^{2}+\frac{1}{6}\left(14n-1\right)x^{2}y^{3}+\frac{9}{9}nx^{3}y^{3},$$ (12)

$$D_{x}^{\alpha }D_{y}^{\alpha }\left(g\left(x,y\right)\right)=2^{\alpha }xy^{2}+3^{\alpha }\left(7n+1\right)xy^{3}+2.2^{\alpha }.2^{\alpha }nx^{2}y^{2}+2^{\alpha }.3^{\alpha }\left(14n-1\right)x^{2}y^{3},$$ (13)

$$\;+9n3^{\alpha }.3^{\alpha }x^{3}y^{3},$$ (14)

$$S_{y}^{\alpha }S_{x}^{\alpha }\left(g\left(x,y\right)\right)=\frac{xy^{2}}{2^{\alpha }}+\frac{7n+1}{3^{\alpha }}xy^{3}+\frac{nx^{2}y^{2}}{2^{\alpha -1}.2^{\alpha }}+\frac{14n-1}{2^{\alpha }.3^{\alpha }}x^{2}y^{3}+\frac{9nx^{3}y^{3}}{3^{\alpha }.3^{\alpha }},$$ (15)

$$S_{y}D_{x}\left(g\left(x,y\right)\right)=\frac{xy^{2}}{2}+\frac{7n+1}{3}xy^{3}+2nx^{2}y^{2}+\frac{2}{3}\left(14n-1\right)x^{2}y^{3}+9nx^{3}y^{3},$$ (16)

$$S_{x}D_{y}\left(g\left(x,y\right)\right)=2xy^{2}+3\left(7n+1\right)xy^{3}+2nx^{2}y^{2}+\frac{3}{2}\left(14n-1\right)x^{2}y^{3}+9nx^{3}y^{3}.$$ (17)

From Table 1, we obtain the following equation:

$$\begin{array}{ll} 1.M_{1}\left(\Gamma \right) & =\left(D_{x}+D_{y}\right)\left(M\left(\Gamma ;x,y\right)\right){|}_{x=y=1}=160n+2.\\ 2.M_{2}\left(\Gamma \right) & =\left(D_{x}D_{y}\right)\left(M\left(\Gamma ;x,y\right)\right){|}_{x=y=1}=194n-1.\\ 3.^{m}M_{2}\left(\Gamma \right) & =\left(S_{x}S_{y}\right)\left(M\left(\Gamma ;x,y\right)\right){|}_{x=y=1}=6.167n+0.67.\\ 4.R_{\alpha }\left(\Gamma \right) & =\left(D_{x}^{\alpha }D_{y}^{\alpha }\right)\left(M\left(\Gamma ;x,y\right)\right){|}_{x=y=1}=2^{\alpha }+3^{\alpha }\left(7n+1\right)+2^{2\alpha +1}n+2^{\alpha }3^{\alpha }\left(14n-1\right)\\ & \;+3^{2\alpha +2}n.\\ 5.RR_{\alpha }\left(\Gamma \right) & =\left(S_{x}^{\alpha }S_{y}^{\alpha }\right)\left(M\left(\Gamma ;x,y\right)\right){|}_{x=y=1}=\frac{1}{2^{\alpha }}+\frac{\left(7n+1\right)}{3^{\alpha }}+\frac{n}{2^{2\alpha -1}}+\frac{\left(14n-1\right)}{2^{\alpha }3^{\alpha }}+\frac{n}{3^{2\alpha -2}}.\\ 6.SDD\left(\Gamma \right) & =\left(D_{x}S_{y}+S_{x}D_{y}\right)\left(M\left(\Gamma ;x,y\right)\right){|}_{x=y=1}=75.67n+2.5. \end{array}$$

Proposition 3.4 *Consider a molecular graph* Γ *for guar gum. Then,*

$$\begin{array}{ll} 1.H\left(\Gamma \right) & =13.1n+0.76.\\ 2.I\left(\Gamma \right) & =37.55n+0.22.\\ 3.AZI\left(\Gamma \right) & =254.125n+3.375. \end{array}$$

Proof: From the edge partitioning of guar gum and using the definition M -polynomial of Γ, we obtain the following equations (Eqs 17–20):

$$g\left(x,y\right)=M\left(\Gamma ;x,y\right)=xy^{2}+\left(7n+1\right)xy^{3}+2nx^{2}y^{2}+\left(14n-1\right)x^{2}y^{3}+9nx^{3}y^{3}.$$

$$I\left(g\left(x,y\right)\right)=x^{3}+\left(7n+1\right)x^{4}+2nx^{4}+2\left(14n-1\right)x^{5}+9nx^{6},$$ (18)

$$S_{x}I\left(g\left(x,y\right)\right)=\frac{1}{3}x^{3}+\frac{\left(7n+1\right)}{4}x^{4}+\frac{1}{2}nx^{4}+\frac{1}{5}\left(14n-1\right)x^{5}+\frac{3}{2}nx^{6},$$ (19)

$$S_{x}ID_{x}D_{y}\left(g\left(x,y\right)\right)=\frac{2}{3}x^{3}+\frac{3\left(7n+1\right)}{4}x^{4}+2nx^{4}+\frac{6}{5}\left(14n-1\right)x^{5}+\frac{27}{2}nx^{6},$$ (20)

$$S_{x}^{3}Q_{-2}ID_{x}^{3}D_{y}^{3}\left(g\left(x,y\right)\right)=8x+\frac{27\left(7n+1\right)}{8}x^{2}+16nx^{2}+8\left(14n-1\right)x^{3}+\frac{9.27.27}{64}nx^{4}.$$ (21)

From Table 1, we obtain the following equation:

$$\begin{array}{ll} 1.H\left(\Gamma \right) & =2\left(S_{x}I\right)\left(M\left(\Gamma ;x,y\right)\right){|}_{x=1}=13.1n+0.76.\\ 2.I\left(\Gamma \right) & =\left(S_{x}ID_{x}D_{y}\right)\left(M\left(\Gamma ;x,y\right)\right){|}_{x=1}=37.55n+0.22.\\ 3.AZI\left(\Gamma \right) & =\left(S_{x}^{3}Q_{-2}ID_{x}^{3}D_{y}^{3}\right)\left(M\left(\Gamma ;x,y\right)\right){|}_{x=1}=254.125n+3.375. \end{array}$$

### 3.2 Results of HPG and CMG molecular graphs

**FIGURE 5:**
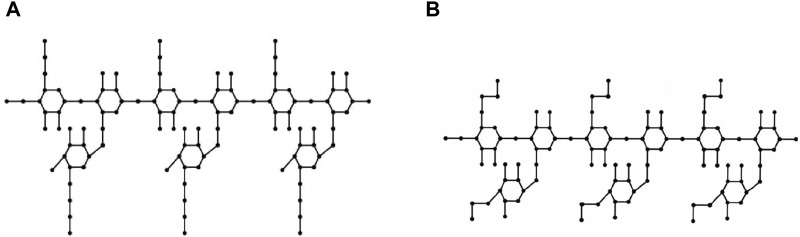
Molecular graph for *n* = 3: **(A)** HPG and **(B)** CMG.

When the chemical derivatives of guar gum, such as HPG and CMG , were developed into molecular graphs. The results for these chemical derivatives were combined, as shown below, because the vertex and edge partitions are similar. Figure 5 shows the molecular graphs of HPG and CMG . From the vertices and edges of HPG and CMG , we obtain the following equation:

$$E_{\left\{i_{0},i_{1}\right\}}=\left\{e=\upsilon \nu \in E\left(CMG\left[m,n\right]\right):d_{\upsilon }=i_{0},d_{\nu }=i_{1}\right\},$$

such that |*E*_{1,2}_| = 2*n* + 1, |*E*_{1,3}_| = 7*n* + 1, |*E*_{2,2}_| = 4*n*, |*E*_{2,3}_| = 14*n* − 1, and |*E*_{3,3}_| = 12*n*.

Theorem 3.6 *Consider a molecular graph* Γ *for hydroxypropyl Guar and carboxymethyl guar. Then,*

$$M\left(CMG;x,y\right)=\left(2n+1\right)xy^{2}+\left(7n+1\right)xy^{3}+4nx^{2}y^{2}+\left(14n-1\right)x^{2}y^{3}+12nx^{3}y^{3}.$$

Proof: From the edge partitioning of HPG and CMG and using the definition M -polynomial of Γ, we obtain the following equation:

$$\begin{array}{ll} M\left(CMG;x,y\right) & =\underset{i_{0}\leq i_{1}}{\sum }m_{i_{0}i_{1}}\left(\Gamma \right)x^{i_{0}}y^{i_{1}}.\\ & =\underset{1\leq 2}{\sum }m_{12}\left(\Gamma \right)x^{1}y^{2}+\underset{1\leq 3}{\sum }m_{13}\left(\Gamma \right)x^{1}y^{3}+\underset{2\leq 2}{\sum }m_{22}\left(\Gamma \right)x^{2}y^{2}+\underset{2\leq 3}{\sum }m_{22}\left(\Gamma \right)x^{2}y^{3}\\ & \;+\underset{3\leq 3}{\sum }m_{33}\left(\Gamma \right)x^{3}y^{3}.\\ & =\underset{uv\in E_{\left\{1,2\right\}}}{\sum }m_{12}\left(\Gamma \right)x^{1}y^{2}+\underset{uv\in E_{\left\{1,3\right\}}}{\sum }m_{13}\left(\Gamma \right)x^{1}y^{3}+\underset{uv\in E_{\left\{2,2\right\}}}{\sum }m_{22}\left(\Gamma \right)x^{2}y^{2}\\ & \;+\underset{uv\in E_{\left\{2,3\right\}}}{\sum }m_{23}\left(\Gamma \right)x^{2}y^{3}+\underset{uv\in E_{\left\{3,3\right\}}}{\sum }m_{33}\left(\Gamma \right)x^{3}y^{3}.\\ & =|E_{\left\{1,2\right\}}x^{1}y^{2}+|E_{\left\{1,3\right\}}|x^{1}y^{3}+|E_{\left\{2,2\right\}}|x^{2}y^{2}+|E_{\left\{2,3\right\}}|x^{2}y^{3}+|E_{\left\{3,3\right\}}|x^{3}y^{3}.\\ & =\left(2n+1\right)xy^{2}+\left(7n+1\right)xy^{3}+4nx^{2}y^{2}+\left(14n-1\right)x^{2}y^{3}+12nx^{3}y^{3}. \end{array}$$

Proposition 3.7 *Consider a molecular graph* Γ *for hydroxypropyl guar and carboxymethyl guar. Then,*

$$\begin{array}{ll} 1.M_{1}\left(\Gamma \right) & =192n+1.\\ 2.M_{2}\left(\Gamma \right) & =233n-1.\\ 3.^{m}M_{2}\left(\Gamma \right) & =7.99n+0.663.\\ 4.R_{\alpha }\left(\Gamma \right) & =2^{\alpha }\left(2n+1\right)+3^{\alpha }\left(7n+1\right)+2^{2\alpha +2}n+2^{\alpha }3^{\alpha }\left(14n-1\right)+4.3^{2\alpha +1}n.\\ 5.RR_{\alpha }\left(\Gamma \right) & =\frac{1}{2^{\alpha }}\left(2n+1\right)+\frac{\left(7n+1\right)}{3^{\alpha }}+\frac{n}{2^{2\alpha -2}}+\frac{\left(14n-1\right)}{2^{\alpha }3^{\alpha }}+\frac{4n}{3^{2\alpha -1}}.\\ 6.SDD\left(\Gamma \right) & =90.66n+3.66. \end{array}$$

Proof: From the edge partitioning of HPG and CMG and using the definition M -polynomial of Γ, we obtain the following equations (Eqs 21–30):

$$\begin{array}{ll} h\left(x,y\right) & =M\left(CMG;x,y\right)=\left(2n+1\right)xy^{2}+\left(7n+1\right)xy^{3}+4nx^{2}y^{2}+\left(14n-1\right)x^{2}y^{3}\\ & \;+12nx^{3}y^{3}, \end{array}$$

$$D_{x}\left(h\left(x,y\right)\right)=\left(2n+1\right)xy^{2}+\left(7n+1\right)xy^{3}+8nx^{2}y^{2}+2\left(14n-1\right)x^{2}y^{3}+36nx^{3}y^{3},$$ (22)

$$D_{y}\left(h\left(x,y\right)\right)=2\left(2n+1\right)xy^{2}+3\left(7n+1\right)xy^{3}+8nx^{2}y^{2}+3\left(14n-1\right)x^{2}y^{3}+36nx^{3}y^{3},$$ (23)

$$D_{x}D_{y}\left(h\left(x,y\right)\right)=2\left(2n+1\right)xy^{2}+3\left(7n+1\right)xy^{3}+16nx^{2}y^{2}+6\left(14n-1\right)x^{2}y^{3}+108nx^{3}y^{3},$$ (24)

$$S_{x}\left(h\left(x,y\right)\right)=\left(2n+1\right)xy^{2}+\left(7n+1\right)xy^{3}+2nx^{2}y^{2}+\frac{1}{2}\left(14n-1\right)x^{2}y^{3}+4nx^{3}y^{3},$$ (25)

$$S_{y}S_{x}\left(h\left(x,y\right)\right)=\frac{\left(2n+1\right)}{2}xy^{2}+\frac{1}{3}\left(7n+1\right)xy^{3}+2nx^{2}y^{2}+\frac{1}{3}\left(14n-1\right)x^{2}y^{3}+4nx^{3}y^{3},$$ (26)

$$D_{x}^{\alpha }D_{y}^{\alpha }\left(h\left(x,y\right)\right)=2^{\alpha }\left(2n+1\right)xy^{2}+3^{\alpha }\left(7n+1\right)xy^{3}+4.2^{\alpha }.2^{\alpha }nx^{2}y^{2},$$ (27)

$$\;+2^{\alpha }.3^{\alpha }\left(14n-1\right)x^{2}y^{3}+12n3^{\alpha }.3^{\alpha }x^{3}y^{3},$$ (28)

$$S_{y}^{\alpha }S_{x}^{\alpha }\left(h\left(x,y\right)\right)=\frac{\left(2n+1\right)xy^{2}}{2^{\alpha }}+\frac{7n+1}{3^{\alpha }}xy^{3}+\frac{4nx^{2}y^{2}}{2^{\alpha }.2^{\alpha }}+\frac{14n-1}{2^{\alpha }.3^{\alpha }}x^{2}y^{3}+\frac{12nx^{3}y^{3}}{3^{\alpha }.3^{\alpha }},$$ (29)

$$S_{y}D_{x}\left(h\left(x,y\right)\right)=\frac{\left(2n+1\right)xy^{2}}{2}+\frac{7n+1}{3}xy^{3}+4nx^{2}y^{2}+\frac{2}{3}\left(14n-1\right)x^{2}y^{3}+12nx^{3}y^{3},$$ (30)

$$S_{x}D_{y}\left(h\left(x,y\right)\right)=2\left(2n+1\right)xy^{2}+3\left(7n+1\right)xy^{3}+4nx^{2}y^{2}+\frac{3}{2}\left(14n-1\right)x^{2}y^{3}+12nx^{3}y^{3}.$$ (31)

From Table 1, we obtain the following equation:

$$\begin{array}{ll} 1.M_{1}\left(\Gamma \right) & =\left(D_{x}+D_{y}\right)\left(M\left(CMG;x,y\right)\right){|}_{x=y=1}=192n+1.\\ 2.M_{2}\left(\Gamma \right) & =\left(D_{x}D_{y}\right)\left(M\left(CMG;x,y\right)\right){|}_{x=y=1}=233n-1.\\ 3.^{m}M_{2}\left(\Gamma \right) & =\left(S_{x}S_{y}\right)\left(M\left(CMG;x,y\right)\right){|}_{x=y=1}=7.99n+0.663.\\ 4.R_{\alpha }\left(\Gamma \right) & =\left(D_{x}^{\alpha }D_{y}^{\alpha }\right)\left(M\left(CMG;x,y\right)\right){|}_{x=y=1}=2^{\alpha }\left(2n+1\right)+3^{\alpha }\left(7n+1\right)+2^{2\alpha +2}n\\ & \;+2^{\alpha }3^{\alpha }\left(14n-1\right)+4.3^{2\alpha +1}n.\\ 5.RR_{\alpha }\left(\Gamma \right) & =\left(S_{x}^{\alpha }S_{y}^{\alpha }\right)\left(M\left(CMG;x,y\right)\right){|}_{x=y=1}=\frac{1}{2^{\alpha }}\left(2n+1\right)+\frac{\left(7n+1\right)}{3^{\alpha }}+\frac{n}{2^{2\alpha -2}}\\ & \;+\frac{\left(14n-1\right)}{2^{\alpha }3^{\alpha }}+\frac{4n}{3^{2\alpha -1}}.\\ 6.SDD\left(\Gamma \right) & =\left(D_{x}S_{y}+S_{x}D_{y}\right)\left(M\left(CMG;x,y\right)\right){|}_{x=y=1}=90.66n+3.66. \end{array}$$

Proposition 3.8 *Consider a molecular graph* Γ *for hydroxypropyl guar and carboxymethyl guar. Then,*

$$\begin{array}{ll} 1.H\left(\Gamma \right) & =16.44n+0.78.\\ 2.I\left(\Gamma \right) & =45.38n+0.22.\\ 3.AZI\left(\Gamma \right) & =320.315n+3.375. \end{array}$$

Proof: From the edge partitioning of HPG and CMG and using the definition M -polynomial of Γ, we obtain the following equations (Eqs 31–34):

$$h\left(x,y\right)=M\left(\Gamma ;x,y\right)=\left(2n+1\right)xy^{2}+\left(7n+1\right)xy^{3}+4nx^{2}y^{2}+\left(14n-1\right)x^{2}y^{3}+12nx^{3}y^{3},$$

$$I\left(h\left(x,y\right)\right)=\left(2n+1\right)x^{3}+\left(7n+1\right)x^{4}+4nx^{4}+\left(14n-1\right)x^{5}+12nx^{6},$$ (32)

$$S_{x}I\left(h\left(x,y\right)\right)=\frac{\left(2n+1\right)}{3}x^{3}+\frac{\left(7n+1\right)}{4}x^{4}+nx^{4}+\frac{1}{5}\left(14n-1\right)x^{5}+2nx^{6},$$ (33)

$$S_{x}ID_{x}D_{y}\left(h\left(x,y\right)\right)=\frac{2}{3}\left(2n+1\right)x^{3}+\frac{3\left(7n+1\right)}{4}x^{4}+4nx^{4}+\frac{6}{5}\left(14n-1\right)x^{5}+18nx^{6},$$ (34)

$$S_{x}^{3}Q_{-2}ID_{x}^{3}D_{y}^{3}\left(h\left(x,y\right)\right)=8\left(2n+1\right)x+\frac{27\left(7n+1\right)}{8}x^{2}+32nx^{2}+8\left(14n-1\right)x^{3},$$ (35)

$$\;+\frac{12.27.27}{64}nx^{4}.$$ (36)

From Table 1, we obtain the following equation:

$$\begin{array}{ll} 1.H\left(\Gamma \right) & =2\left(S_{x}I\right)\left(M\left(CMG;x,y\right)\right){|}_{x=1}=16.44n+0.78.\\ 2.I\left(\Gamma \right) & =\left(S_{x}ID_{x}D_{y}\right)\left(M\left(CMG;x,y\right)\right){|}_{x=1}=45.38n+0.22.\\ 3.AZI\left(\Gamma \right) & =\left(S_{x}^{3}Q_{-2}ID_{x}^{3}D_{y}^{3}\right)\left(M\left(CMG;x,y\right)\right){|}_{x=1}=320.315n+3.375. \end{array}$$

### 3.3 Results for the CMHPG molecular graph

**FIGURE 6:**
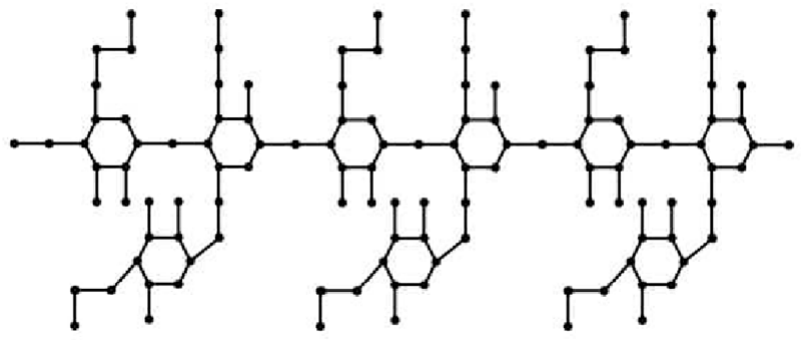
Molecular graph of CMHPG for *n* = 3.

In the carboxymethyl hydroxypropyl guar molecular graph shown in Figure 6, for the vertices and edges, we obtain the following equation:

$$E_{\left\{i_{0},i_{1}\right\}}=\left\{e=\upsilon \nu \in E\left(CMHPG\left[m,n\right]\right):d_{\upsilon }=i_{0},d_{\nu }=i_{1}\right\},$$

such that |*E*_{1,2}_| = 3*n* + 1, |*E*_{1,3}_| = 6*n* + 1, |*E*_{2,2}_| = 5*n*, |*E*_{2,3}_| = 15*n* − 1, and |*E*_{3,3}_| = 12*n*.

Theorem 3.10 *Consider a molecular graph* Γ *for carboxymethyl hydroxypropyl guar. Then,*

$$M\left(CMHPG;x,y\right)=\left(3n+1\right)xy^{2}+\left(6n+1\right)xy^{3}+5nx^{2}y^{2}+\left(15n-1\right)x^{2}y^{3}+12nx^{3}y^{3}.$$

Proof: From the edge partitioning of CMHPG and using the definition M -polynomial of Γ, we obtain the following equation:

$$\begin{array}{ll} M\left(CMHPG;x,y\right) & =\underset{i_{0}\leq i_{1}}{\sum }m_{i_{0}j_{1}}\left(\Gamma \right)x^{i_{0}}y^{i_{1}}.\\ & =\underset{1\leq 2}{\sum }m_{12}\left(\Gamma \right)x^{1}y^{2}+\underset{1\leq 3}{\sum }m_{13}\left(\Gamma \right)x^{1}y^{3}+\underset{2\leq 2}{\sum }m_{22}\left(\Gamma \right)x^{2}y^{2}+\underset{2\leq 3}{\sum }m_{22}\left(\Gamma \right)x^{2}y^{3}\\ & \;+\underset{3\leq 3}{\sum }m_{33}\left(\Gamma \right)x^{3}y^{3}. \end{array}$$

$$\begin{array}{ll} & =\underset{uv\in E_{\left\{1,2\right\}}}{\sum }m_{12}\left(\Gamma \right)x^{1}y^{2}+\underset{uv\in E_{\left\{1,3\right\}}}{\sum }m_{13}\left(\Gamma \right)x^{1}y^{3}+\underset{uv\in E_{\left\{2,2\right\}}}{\sum }m_{22}\left(\Gamma \right)x^{2}y^{2}\\ & \;+\underset{uv\in E_{\left\{2,3\right\}}}{\sum }m_{23}\left(\Gamma \right)x^{2}y^{3}+\underset{uv\in E_{\left\{3,3\right\}}}{\sum }m_{33}\left(\Gamma \right)x^{3}y^{3}.\\ & =|E_{\left\{1,2\right\}}x^{1}y^{2}+|E_{\left\{1,3\right\}}|x^{1}y^{3}+|E_{\left\{2,2\right\}}|x^{2}y^{2}+|E_{\left\{2,3\right\}}|x^{2}y^{3}+|E_{\left\{3,3\right\}}|x^{3}y^{3}.\\ & =\left(3n+1\right)xy^{2}+\left(6n+1\right)xy^{3}+5nx^{2}y^{2}+\left(15n-1\right)x^{2}y^{3}+12nx^{3}y^{3}. \end{array}$$

Proposition 3.11 *Consider a molecular graph* Γ *for carboxymethyl hydroxypropyl guar. Then,*

$$\begin{array}{ll} 1.M_{1}\left(\Gamma \right) & =200n+2.\\ 2.M_{2}\left(\Gamma \right) & =242n-1.\\ 3.^{m}M_{2}\left(\Gamma \right) & =8.58n+0.663.\\ 4.R_{\alpha }\left(\Gamma \right) & =2^{\alpha }\left(3n+1\right)+3^{\alpha }\left(6n+1\right)+5n.2^{2\alpha }+2^{\alpha }3^{\alpha }\left(15n-1\right)+12n.3^{2\alpha }.\\ 5.RR_{\alpha }\left(\Gamma \right) & =\frac{1}{2^{\alpha }}\left(3n+1\right)+\frac{\left(6n+1\right)}{3^{\alpha }}+\frac{5n}{2^{2\alpha }}+\frac{\left(15n-1\right)}{2^{\alpha }3^{\alpha }}+\frac{12n}{3^{2\alpha }}.\\ 6.SDD\left(\Gamma \right) & =106.03n+3.66. \end{array}$$

Proof: From the edge partitioning of CMHPG and using the definition M -polynomial of Γ, we obtain the following equations (Eqs 35–43):

$$\begin{array}{ll} J\left(x,y\right) & =M\left(CMHPG;x,y\right)=\left(3n+1\right)xy^{2}+\left(6n+1\right)xy^{3}+4nx^{2}y^{2}+\left(15n-1\right)x^{2}y^{3}\\ & \;+12nx^{3}y^{3}, \end{array}$$

$$D_{x}\left(J\left(x,y\right)\right)=\left(3n+1\right)xy^{2}+\left(6n+1\right)xy^{3}+10nx^{2}y^{2}+2\left(15n-1\right)x^{2}y^{3}+36nx^{3}y^{3},$$ (37)

$$D_{y}\left(J\left(x,y\right)\right)=2\left(3n+1\right)xy^{2}+3\left(6n+1\right)xy^{3}+10nx^{2}y^{2}+3\left(15n-1\right)x^{2}y^{3}+36nx^{3}y^{3},$$ (38)

$$D_{x}D_{y}\left(J\left(x,y\right)\right)=2\left(3n+1\right)xy^{2}+3\left(6n+1\right)xy^{3}+20nx^{2}y^{2}+6\left(15n-1\right)x^{2}y^{3}+108nx^{3}y^{3},$$ (39)

$$S_{x}\left(J\left(x,y\right)\right)=\left(3n+1\right)xy^{2}+\left(6n+1\right)xy^{3}+\frac{5}{2}nx^{2}y^{2}+\frac{1}{2}\left(15n-1\right)x^{2}y^{3}+4nx^{3}y^{3},$$ (40)

$$S_{y}S_{x}\left(J\left(x,y\right)\right)=\frac{\left(3n+1\right)}{2}xy^{2}+\frac{1}{3}\left(6n+1\right)xy^{3}+\frac{5}{4}nx^{2}y^{2}+\frac{1}{6}\left(15n-1\right)x^{2}y^{3}+\frac{4}{3}nx^{3}y^{3},$$ (41)

$$D_{x}^{\alpha }D_{y}^{\alpha }\left(J\left(x,y\right)\right)=2^{\alpha }\left(3n+1\right)xy^{2}+3^{\alpha }\left(6n+1\right)xy^{3}+5.2^{\alpha }.2^{\alpha }nx^{2}y^{2}+2^{\alpha }.3^{\alpha }\left(15n-1\right)x^{2}y^{3},$$ (42)

$$\;+12n3^{\alpha }.3^{\alpha }x^{3}y^{3},$$ (43)

$$S_{y}^{\alpha }S_{x}^{\alpha }\left(J\left(x,y\right)\right)=\frac{\left(3n+1\right)xy^{2}}{2^{\alpha }}+\frac{6n+1}{3^{\alpha }}xy^{3}+\frac{5nx^{2}y^{2}}{2^{\alpha }.2^{\alpha }}+\frac{15n-1}{2^{\alpha }.3^{\alpha }}x^{2}y^{3}+\frac{12nx^{3}y^{3}}{3^{\alpha }.3^{\alpha }},$$ (44)

$$S_{y}D_{x}\left(J\left(x,y\right)\right)=\frac{\left(3n+1\right)xy^{2}}{2}+\frac{6n+1}{3}xy^{3}+5nx^{2}y^{2}+\frac{2}{3}\left(15n-1\right)x^{2}y^{3}+12nx^{3}y^{3},$$ (45)

$$S_{x}D_{y}\left(J\left(x,y\right)\right)=2\left(3n+1\right)xy^{2}+3\left(6n+1\right)xy^{3}+5nx^{2}y^{2}+\frac{3}{2}\left(15n-1\right)x^{2}y^{3}+12nx^{3}y^{3}.$$ (46)

From Table 1, we obtain the following equation:

$$\begin{array}{ll} 1.M_{1}\left(\Gamma \right) & =\left(D_{x}+D_{y}\right)\left(M\left(CMHPG;x,y\right)\right){|}_{x=y=1}=200n+2.\\ 2.M_{2}\left(\Gamma \right) & =\left(D_{x}D_{y}\right)\left(M\left(CMHPG;x,y\right)\right){|}_{x=y=1}=242n-1.\\ 3.^{m}M_{2}\left(\Gamma \right) & =\left(S_{x}S_{y}\right)\left(M\left(CMHPG;x,y\right)\right){|}_{x=y=1}=8.58n+0.663.\\ 4.R_{\alpha }\left(\Gamma \right) & =\left(D_{x}^{\alpha }D_{y}^{\alpha }\right)\left(M\left(CMHPG;x,y\right)\right){|}_{x=y=1}=2^{\alpha }\left(3n+1\right)+3^{\alpha }\left(6n+1\right)+5n.2^{2\alpha }\\ & \;+2^{\alpha }3^{\alpha }\left(15n-1\right)+12n.3^{2\alpha }.\\ 5.RR_{\alpha }\left(\Gamma \right) & =\left(S_{x}^{\alpha }S_{y}^{\alpha }\right)\left(M\left(CMHPG;x,y\right)\right){|}_{x=y=1}=\frac{1}{2^{\alpha }}\left(3n+1\right)+\frac{\left(6n+1\right)}{3^{\alpha }}+\frac{5n}{2^{2\alpha }}\\ & \;+\frac{\left(15n-1\right)}{2^{\alpha }3^{\alpha }}+\frac{12n}{3^{2\alpha }}.\\ 6.SDD\left(\Gamma \right) & =\left(D_{x}S_{y}+S_{x}D_{y}\right)\left(M\left(CMHPG;x,y\right)\right){|}_{x=y=1}=106.03n+3.66. \end{array}$$

Proposition 3.12 *Consider a molecular graph* Γ *for carboxymethyl hydroxypropyl guar. Then,*

1. *H*(Γ) = 17.5*n* + 0.76
2. *I*(Γ) = 47.5*n* + 0.22
3. *AZI*(Γ) = 340.95*n* + 3.4

Proof: From the edge partitioning of CMHPG and using the definition M -polynomial of Γ, we obtain the following equations (Eqs 44–47):

$$\begin{array}{ll} J\left(x,y\right) & =M\left(CMPHG;x,y\right)=\left(3n+1\right)xy^{2}+\left(6n+1\right)xy^{3}+5nx^{2}y^{2}+\left(15n-1\right)x^{2}y^{3}\\ & \;+12nx^{3}y^{3}, \end{array}$$

$$I\left(J\left(x,y\right)\right)=\left(3n+1\right)x^{3}+\left(6n+1\right)x^{4}+5nx^{4}+\left(15n-1\right)x^{5}+12nx^{6},$$ (47)

$$S_{x}I\left(J\left(x,y\right)\right)=\frac{\left(3n+1\right)}{3}x^{3}+\frac{\left(6n+1\right)}{4}x^{4}+\frac{5}{4}nx^{4}+\frac{1}{5}\left(15n-1\right)x^{5}+2nx^{6},$$ (48)

$$S_{x}ID_{x}D_{y}\left(J\left(x,y\right)\right)=\frac{2}{3}\left(3n+1\right)x^{3}+\frac{3\left(6n+1\right)}{4}x^{4}+5nx^{4}+\frac{6}{5}\left(15n-1\right)x^{5}+18nx^{6},$$ (49)

$$S_{x}^{3}Q_{-2}ID_{x}^{3}D_{y}^{3}\left(J\left(x,y\right)\right)=8\left(3n+1\right)x+\frac{27\left(6n+1\right)}{8}x^{2}+40nx^{2}+8\left(15n-1\right)x^{3},$$ (50)

$$\;+\frac{12.27.27}{64}nx^{4}.$$ (51)

From Table 1, we obtain the following equation:

$$\begin{array}{ll} 1.H\left(\Gamma \right) & =2\left(S_{x}I\right)\left(M\left(CMHPG;x,y\right)\right){|}_{x=1}=17.5n+0.76.\\ 2.I\left(\Gamma \right) & =\left(S_{x}ID_{x}D_{y}\right)\left(M\left(CMHPG;x,y\right)\right){|}_{x=1}=47.5n+0.22.\\ 3.AZI\left(\Gamma \right) & =\left(S_{x}^{3}Q_{-2}ID_{x}^{3}D_{y}^{3}\right)\left(M\left(CMHPG;x,y\right)\right){|}_{x=1}=340.95n+3.4. \end{array}$$

## 4 Molecular structures and computations of topological indices for different carbohydrates

Carbohydrates are organic molecules made up of carbon, hydrogen, and oxygen. They come in three main types: monosaccharides, disaccharides, and polysaccharides. These sugars or polymers can be aldehydes or ketones and serve various purposes in various species. Exoskeletons, found on arthropods, are made of chitin, a nitrogen-containing polymer. Guar gum, a non-ionic polysaccharide derived from the cluster bean endosperm, is a new agrochemical. Polysaccharides are polymers formed when several monomer units are linked together through condensation. They are the most common type of biomolecules and are found in various sources, including algal, plant, microbial, and animal sources. The structural parameters of polysaccharides typically define its chemical compositions, molecular weights, molecular structure, degree of substitution, viscosity, solubility, and particle size, contributing to their structural and chemical properties. These parameters collectively determine the functionality and performance of guar gum in various applications, making it a versatile ingredient in numerous industries. They are highly stable, secure, non-toxic, hydrophilic, and biodegradable as natural biomaterials.

**FIGURE 7:**
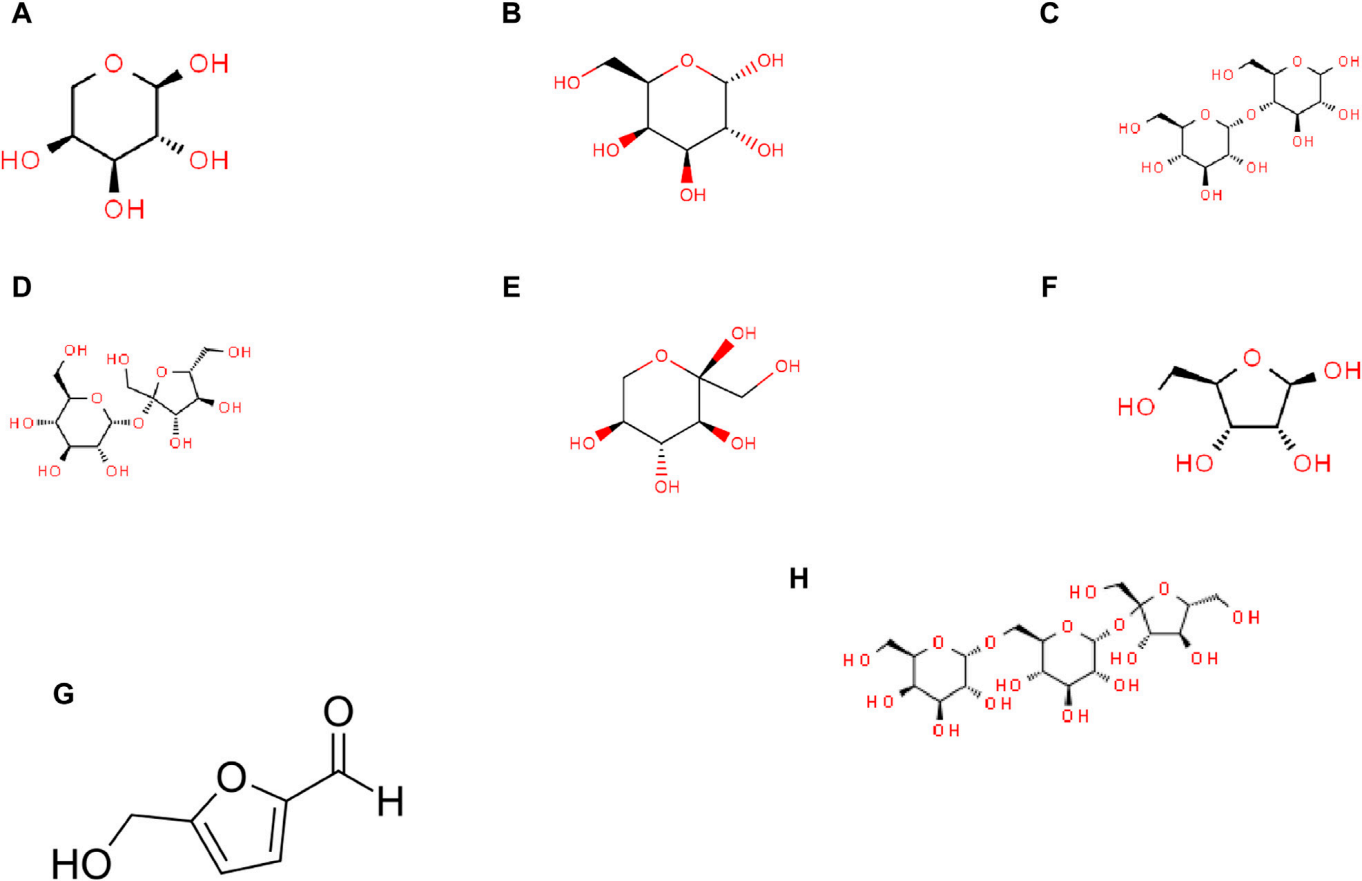
Molecular structures of carbohydrates: **(A)** arabinose, **(B)** galactose, **(C)** maltose, **(D)** sucrose, **(E)** sorbose, **(F)** ribose, **(G)** hydroxymethyl furfural (HMF), and **(H)** raffinose.

This section examines eight carbohydrate molecules: arabinose, galactose, maltose, sucrose, sorbose, ribose, hydroxymethyl furfural, and raffinose. Their molecular structures are depicted in Figure 7, and their physical and chemical properties are listed in Table 2. Table 3 includes consideration of topological indices through vertex and edge partitioning, as defined in Tables 4–11.

**TABLE 2:** Properties of aforementioned structures.

Structure	Density	Boiling Point (BP)	Melting Point (MP)	Molecular Weight (MW)	Water Solubility (WS)
Unit	g/cm^3^	°C	°C	g/mol	g/L
Arabinose	1.585	415.5	164	150.13	834
Galactose	1.5	232.96	168	180.156	650
Maltose	1.54	397.76	160	342.297	310
Sucrose	1.587	697.1	186	342.30	200
Sorbose	1.65	551.7	165	180	550
Ribose	1.5	415.5	95	150.13	100
HMP	1.29	114	30	126.11	83
Raffinose	884.8	363.0	81	504.44	229.8

**TABLE 3:** Computation of topological indices.

Structure	*H*	*I*	*AZI*	*M*_1_	*M*_2_	^*m*^ *M*_2_	*SDD*
Arabinose	4.3	10.9	71.6719	48	55	2.25	25.6667
Galactose	5.2	13.2667	91.0625	59	68	2.7778	30.3333
Maltose	10.2	27.4333	191.375	118	143	5.2222	58.3333
Sucrose	10.4333	27.05	192.6094	116	138	5.4444	57.6667
Sorbose	5.0875	12.2976	89.1006	60	71	2.7222	32
Ribose	4.3667	11.0167	66.4375	48	56	2.3333	25
HMP	4.9	10.2	80	46	49	2.25	25
Raffinose	14.3857	39.0143	271.3396	169	205	7.275	83.25

**TABLE 4:** Vertex and edge partitioning of arabinose.

(*d*_*υ*_, *d*_*ν*_)	Frequency
(1,3)	4
(2,2)	1
(2,3)	2
(3,3)	3

**TABLE 5:** Vertex and edge partitioning of galactose.

(*d*_*υ*_, *d*_*ν*_)	Frequency
(1,2)	1
(1,3)	4
(2,3)	3
(3,3)	4

**TABLE 6:** Vertex and edge partitioning of maltose.

(*d*_*υ*_, *d*_*ν*_)	Frequency
(1,2)	2
(1,3)	6
(2,3)	8
(3,3)	8

**TABLE 7:** Vertex and edge partitioning of sucrose.

(*d*_*υ*_, *d*_*ν*_)	Frequency
(1,2)	3
(1,3)	5
(2,3)	9
(3,3)	7

**TABLE 8:** Vertex and edge partitioning of sorbose.

(*d*_*υ*_, *d*_*ν*_)	Frequency
(1,2)	1
(1,3)	3
(1,4)	1
(2,2)	1
(2,3)	1
(2,4)	2
(3,3)	2
(3,4)	1

**TABLE 9:** Vertex and edge partitioning of ribose.

(*d*_*υ*_, *d*_*ν*_)	Frequency
(1,2)	1
(1,3)	3
(2,3)	3
(3,3)	3

**TABLE 10:** Vertex and edge partitioning of HMP.

(*d*_*υ*_, *d*_*ν*_)	Frequency
(1,2)	3
(1,3)	1
(2,3)	6

**TABLE 11:** Vertex and edge partitioning of raffinose.

(*d*_*υ*_, *d*_*ν*_)	Frequency
(1,2)	3
(1,3)	8
(2,2)	1
(2,3)	9
(2,4)	3
(3,3)	9
(3,4)	1

## 5 Regression models

This section discusses linear, quadratic, and logarithmic regression models. Linear regression predicts the value of one variable based on another, while quadratic models modify variables. Log-regression models linearize variables and test the significance level between topological indices and molecular structures. The mathematical expressions for the linear, quadratic, and logarithmic regression models are as follows:

$$P\;=\;A+B\left(TI\right),$$

$$P\;=\;A+B\left(TI\right)+C{\left(TI\right)}^{2},$$

$$P\;=\;A+Bln\left(TI\right).$$

where P is the property of the molecular structure, *A* is the constant, *B* and *C* are the regression coefficients, and TI is the topological index. The results of these three regression models for the aforementioned indices are calculated using SPSS statistical software, which are depicted in Tables 12–18.

**TABLE 12:** Statistical parameters for *H*(Γ).

Model	r	*r* ^2^	F	P
Linear regression model				
Density = 9.133–0.196 [*H*(Γ)]	0.222	0.049	0.312	0.597
BP = 506.288–3.227 [*H*(Γ)]	0.211	0.045	0.280	0.616
MP = 118.582 + 0.950 [*H*(Γ)]	0.276	0.076	0.495	0.508
MW = 243.839 + 0.235 [*H*(Γ)]	0.028	0.001	0.005	0.947
WS = 293.061 + 5.979 [*H*(Γ)]	0.338	0.114	0.775	0.413
Quadratic regression model				
Density = 0.031 [*H*(Γ)]^2^-1.944 [*H*(Γ)]+19.951	0.394	0.155	0.460	0.656
BP = −1.211 [*H*(Γ)]^2^ + 65.982 [*H*(Γ)]+78.097	0.768	0.590	3.604	0.107
MP = −0.012 [*H*(Γ)]^2^ + 1.623 [*H*(Γ)]+114.412	0.278	0.077	0.209	0.818
MW = −0.885 [*H*(Γ)]^2^ + 50.800 [*H*(Γ)]-69.001	0.988	0.975	99.181	0.0000
WS = 0.660 [*H*(Γ)]^2^-31.903 [*H*(Γ)]+526.307	0.493	0.243	0.803	0.498
Logarithmic regression model				
Density = 18.558–5.507ln [*H*(Γ)]	0.333	0.111	0.747	0.421
BP = 441.473 + 10.184ln [*H*(Γ)]	0.035	0.001	0.007	0.934
MP = 89.682 + 19.018ln [*H*(Γ)]	0.293	0.086	0.563	0.481
MW = 125.979 + 55.510ln [*H*(Γ)]	0.352	0.124	0.848	0.393
WS = 234.016 + 62.235ln [*H*(Γ)]	0.196	0.037	0.230	0.648

**TABLE 13:** Statistical parameters for *I*(Γ).

Model	r	*r* ^2^	F	P
Linear regression model				
Density = 15.633–0.514 [*I*(Γ)]	0.310	0.096	0.637	0.455
BP = 110.644 + 20.004 [*I*(Γ)]	0.691	0.478	5.486	0.058
MP = 108.224 + 1.298 [*I*(Γ)]	0.199	0.040	0.248	0.636
MW = −18.121 + 15.020 [*I*(Γ)]	0.949	0.901	54.429	0.0000
WS = 549.858–10.212 [*I*(Γ)]	0.315	0.099	0.659	0.448
Quadratic regression model				
Density = 0.127 [*I*(Γ)]^2^-5.501 [*I*(Γ)]+55.997	0.347	0.120	0.342	0.726
BP = 1.585 [*I*(Γ)]^2^-42.153 [*I*(Γ)]+613.760	0.700	0.490	2.404	0.186
MP = −2.580 [*I*(Γ)]^2^ + 102.476 [*I*(Γ)]-710.745	0.834	0.696	5.720	0.051
MW = 1.135 [*I*(Γ)]^2^-29.515 [*I*(Γ)]+342.360	0.960	0.922	29.653	0.002
WS = −6.853 [*I*(Γ)]^2^ + 258.578 [*I*(Γ)]-1625.811	0.534	0.285	0.998	0.432
Logarithmic regression model				
Density = 33.404–9.682ln [*I*(Γ)]	0.319	0.102	0.679	0.441
BP = −546.406 + 364.277ln [*I*(Γ)]	0.688	0.474	5.404	0.059
MP = 48.517 + 29.792ln [*I*(Γ)]	0.250	0.063	0.401	0.550
MW = −508.481 + 272.441ln [*I*(Γ)]	0.942	0.887	46.901	0.0000
WS = 832.635–166.978ln [*I*(Γ)]	0.281	0.079	0.516	0.500

**TABLE 14:** Statistical parameters for *AZI*(Γ).

Model	r	*r* ^2^	F	P
Linear regression model				
Density = 14.748–0.062 [*AZI*(Γ)]	0.355	0.112	0.760	0.417
BP = 143.262 + 2.431 [*AZI*(Γ)]	0.752	0.566	7.830	0.031
MP = 128.852 + 0.017 [*AZI*(Γ)]	0.024	0.001	0.003	0.956
MW = 15.678 + 1.754 [*AZI*(Γ)]	0.993	0.987	443.929	0.0000
WS = 525.992–1.186 [*AZI*(Γ)]	0.327	0.107	0.720	0.429
Quadratic regression model				
Density = 0.001 [*AZI*(Γ)]^2^-0.423 [*AZI*(Γ)]+37.145	0.452	0.204	0.642	0.565
BP = 0.015 [*AZI*(Γ)]^2^-2.284 [*AZI*(Γ)]+436.166	0.786	0.618	4.043	0.090
MP = −0.009 [*AZI*(Γ)]^2^ + 2.815 [*AZI*(Γ)]-44.988	0.601	0.361	1.411	0.327
MW = 0.002 [*AZI*(Γ)]^2^ + 1.081 [*AZI*(Γ)]+57.517	0.995	0.990	252.564	0.0000
WS = −0.001 [*AZI*(Γ)]^2^-0.726 [*AZI*(Γ)]+497.430	0.328	0.108	0.301	0.752
Logarithmic regression model				
Density = 56.222–10.460ln [*AZI*(Γ)]	0.398	0.159	1.130	0.329
BP = −1074.714 + 323.998ln [*AZI*(Γ)]	0.707	0.501	6.012	0.050
MP = 76.730 + 11.456ln [*AZI*(Γ)]	0.111	0.012	0.075	0.793
MW = −910.201 + 243.707ln [*AZI*(Γ)]	0.973	0.947	107.701	0.0000
WS = 1123.059–158.678ln [*AZI*(Γ)]	0.309	0.095	0.633	0.457

**TABLE 15:** Statistical parameters for *M*_1_(Γ).

Model	r	*r* ^2^	F	P
Linear regression model				
Density = 1.375 + 0.002 [*M*_1_(Γ)]	0.684	0.467	5.261	0.062
BP = 115.600 + 4.200 [*M*_1_(Γ)]	0.781	0.611	9.413	0.022
MP = 127.371 + 0.045 [*M*_1_(Γ)]	0.037	0.001	0.008	0.930
MW = 3.754 + 2.934 [*M*_1_(Γ)]	0.999	0.997	2200.450	0.0000
WS = 522.419–1.844 [*M*_1_(Γ)]	0.306	0.094	0.619	0.461
Quadratic regression model				
Density = −0.001 [*M*_1_(Γ)]^2^ + 0.0000 [*M*_1_(Γ)]+1.519	0.706	0.498	2.482	0.178
BP = 0.536 [*M*_1_(Γ)]^2^ + 0.018 [*M*_1_(Γ)]+262.656	0.789	0.622	4.119	0.088
MP = −0.026 [*M*_1_(Γ)]^2^ + 5.434 [*M*_1_(Γ)]-88.890	0.705	0.497	2.468	0.180
MW = 0.002 [*M*_1_(Γ)]^2^ + 2.613 [*M*_1_(Γ)]+16.660	0.999	0.998	1030.398	0.0000
WS = −0.010 [*M*_1_(Γ)]^2^ + 0.252 [*M*_1_(Γ)]+438.332	0.311	0.097	0.267	0.776
Logarithmic regression model				
Density = 0.729 + 0.193ln [*M*_1_(Γ)]	0.664	0.441	4.735	0.072
BP = −1125.431 + 369.772ln [*M*_1_(Γ)]	0.763	0.582	8.360	0.028
MP = 59.381 + 16.694ln [*M*_1_(Γ)]	0.153	0.023	0.144	0.717
MW = −875.480 + 261.181ln [*M*_1_(Γ)]	0.986	0.972	204.691	0.0000
WS = 1020.733–151.515ln [*M*_1_(Γ)]	0.279	0.078	0.505	0.504

**TABLE 16:** Statistical parameters for *M*_2_(Γ).

Model	r	*r* ^2^	F	P
Linear regression model				
Density = 1.380 + 0.002 [*M*_2_(Γ)]	0.698	0.487	5.703	0.054
BP = 129.011 + 3.415 [*M*_2_(Γ)]	0.789	0.622	9.882	0.020
MP = 126.188 + 0.050 [*M*_2_(Γ)]	0.052	0.003	0.016	0.903
MW = 15.142 + 2.365 [*M*_2_(Γ)]	0.999	0.999	4276.696	0.0000
WS = 509.284–1.425 [*M*_2_(Γ)]	0.293	0.086	0.565	0.481
Quadratic regression model				
Density = 0.0000 [*M*_2_(Γ)]^2^ + 0.000 [*M*_2_(Γ)]+1.473	0.710	0.504	2.538	0.173
BP = 0.008 [*M*_2_(Γ)]^2^ + 1.463 [*M*_2_(Γ)]+220.317	0.792	0.628	4.220	0.084
MP = −0.018 [*M*_2_(Γ)]^2^ + 4.370 [*M*_2_(Γ)]-75.841	0.748	0.559	3.169	0.129
MW = 0.001 [*M*_2_(Γ)]^2^ + 2.122 [*M*_2_(Γ)]+26.541	0.999	0.999	2268.338	0.0000
WS = −0.011 [*M*_2_(Γ)]^2^ + 1.174 [*M*_2_(Γ)]+387.701	0.307	0.094	0.260	0.781
Logarithmic regression model				
Density = 0.714 + 0.190ln [*M*_2_(Γ)]	0.690	0.476	5.459	0.058
BP = −1120.210 + 355.799ln [*M*_2_(Γ)]	0.777	0.604	9.134	0.023
MP = 44.322 + 19.499ln [*M*_2_(Γ)]	0.189	0.036	0.223	0.654
MW = −849.292 + 246.357ln [*M*_2_(Γ)]	0.983	0.967	176.423	0.0000
WS = 947.654–129.845ln [*M*_2_(Γ)]	0.253	0.064	0.410	0.546

**TABLE 17:** Statistical parameters for ^*m*^ *M*_2_(Γ).

Model	r	*r* ^2^	F	P
Linear regression model				
Density = 1.367 + 0.050 [^*m*^ *M*_2_(Γ)]	0.665	0.443	4.770	0.072
BP = 90.113 + 98.384 [^*m*^ *M*_2_(Γ)]	0.780	0.609	9.335	0.022
MP = 125.904 + 1.375 [^*m*^ *M*_2_(Γ)]	0.048	0.02	0.014	0.909
MW = −13.971 + 68.719 [^*m*^ *M*_2_(Γ)]	0.997	0.993	905.950	0.0000
WS = 543.926–45.903 [^*m*^ *M*_2_(Γ)]	0.325	0.105	0.706	0.433
Quadratic regression model				
Density = 0.013 [^*m*^ *M*_2_(Γ)]^2^-0.072 [^*m*^ *M*_2_(Γ)]+1.593	0.704	0.495	2.451	0.181
BP = −52.840 [^*m*^ *M*_2_(Γ)]^2^ + 16.606 [^*m*^ *M*_2_(Γ)]+369.528	0.798	0.637	4.393	0.079
MP = −14.861 [^*m*^ *M*_2_(Γ)]^2^ + 136.708 [^*m*^ *M*_2_(Γ)]-124.149	0.674	0.454	2.080	0.220
MW = 2.326 [^*m*^ *M*_2_(Γ)]^2^ + 47.542 [^*m*^ *M*_2_(Γ)]+25.157	0.998	0.995	528.816	0.0000
WS = −3.924 [^*m*^ *M*_2_(Γ)]^2^-10.168 [^*m*^ *M*_2_(Γ)]+477.899	0.324	0.107	0.298	0.754
Logarithmic regression model				
Density = 1.313 + 0.199ln [^*m*^ *M*_2_(Γ)]	0.640	0.409	4.154	0.088
BP = −17.267 + 391.087ln [^*m*^ *M*_2_(Γ)]	0.754	0.569	7.926	0.031
MP = 110.702 + 16.608ln [^*m*^ *M*_2_(Γ)]	0.142	0.020	0.124	0.737
MW = −95.851 + 278.575ln [^*m*^ *M*_2_(Γ)]	0.983	0.967	177.008	0.0000
WS = 589.578–178.852ln [^*m*^ *M*_2_(Γ)]	0.303	0.095	0.627	0.459

**TABLE 18:** Statistical parameters for *SDD*(Γ).

Model	r	*r* ^2^	F	P
Linear regression model				
Density = 1.361 + 0.005 [*SDD*(Γ)]	0.688	0.473	5.388	0.059
BP = 88.568 + 8.898 [*SDD*(Γ)]	0.784	0.615	9.587	0.021
MP = 127.401 + 0.088 [*SDD*(Γ)]	0.035	0.001	0.007	0.935
MW = −14.123 + 6.193 [*SDD*(Γ)]	0.998	0.997	1803.041	0.0000
WS = 530.836–3.824 [*SDD*(Γ)]	0.300	0.090	0.596	0.470
Quadratic regression model				
Density = 0.0000 [*SDD*(Γ)]^2^-0.003 [*SDD*(Γ)]+1.521	0.709	0.503	2.529	0.174
BP = 0.077 [*SDD*(Γ)]^2^ + 0.960 [*SDD*(Γ)]+253.757	0.791	0.626	9.194	0.085
MP = −0.115 [*SDD*(Γ)]^2^ + 11.835 [*SDD*(Γ)]-117.049	0.701	0.492	2.420	0.184
MW = 0.005 [*SDD*(Γ)]^2^ + 5.678 [*SDD*(Γ)]-3.409	0.998	0.997	789.458	0.0000
WS = −0.053 [*SDD*(Γ)]^2^ + 1.594 [*SDD*(Γ)]+418.090	0.307	0.094	0.261	0.780
Logarithmic regression model				
Density = 0.798 + 0.209ln [*SDD*(Γ)]	0.667	0.445	4.819	0.071
BP = −988.425 + 399.150ln [*SDD*(Γ)]	0.765	0.585	8.465	0.027
MP = 69.491 + 16.942ln [*SDD*(Γ)]	0.144	0.021	0.128	0.733
MW = −776.633 + 281.361ln [*SDD*(Γ)]	0.986	0.973	213.264	0.0000
WS = 951.942–160.064ln [*SDD*(Γ)]	0.274	0.075	0.485	0.512

## 6 Discussion

A regression model is a statistical technique used to predict the value of a continuous target variable based on input features. Four metrics are crucial in regression analysis: correlation coefficient (*r*), R-squared (*r* ^2^), F-statistic, and *p*-value. The R-squared value measures how well the independent variables predict the dependent variable, with a higher R-squared indicating a stronger linear relationship. The F-statistic tests the overall significance of the model, with a significant F-statistic indicating a non-zero effect of at least one independent variable on the dependent variable. A small *p*-value indicates statistical significance, indicating that at least one independent variable has a significant effect on the dependent variable.

This study employs linear, quadratic, and logarithmic regression models to predict five physical and chemical properties of various carbohydrates, considering topological indices as independent and properties as dependent variables. It has been observed that two properties, namely, molecular weight and boiling point, give the best predicted value by each regression model (as the value of *R* is greater than 0.68 in each case except for linear and logarithmic regressions of the topological index *H*). Linear, quadratic, and logarithmic models and their four important metric values are shown in Tables 12–18. The study focuses on the significance of topological descriptors in predicting the molecular structures of gaur gum and its derivatives, which are crucial in predicting the molecular weight of carbohydrates. Quantitative structure–property relationship (QSPR) methodology is a powerful approach used in the field of drug design, material science, environmental chemistry, cheminformatics, and computational chemistry. QSPR methodology focuses on establishing mathematical relationships between the chemical structure of compounds and their properties, allowing for the prediction of properties based on molecular features. One notable difference is that QSPR specifically targets physical and chemical properties of compounds, while QSAR often focuses on biological activities. Additionally, molecular modeling techniques may involve more complex simulations and calculations to predict molecular behavior. Finally, QSPR methodology offers a systematic and quantitative approach to predict physicochemical properties of chemical compounds based on their molecular structure, distinct from other methodologies such as QSAR, MD simulation, DFT, machine learning models, and hybrid QSAR/QSPR models, each with its own advantages and limitations depending on the specific application and research goals.

## 7 Conclusion

This study focuses on the analysis of the polysaccharide guar gum and its chemical variants, namely, HPG , CMG , and CMHPG . Initially, molecular graphs are used to represent these polysaccharides, and vertex and edge partitions are defined. The closed form of the M -polynomial is then computed for these molecular graphs, using various topological indices such as Zagreb indices, Randi $\overset{´}{c}$ index, inverse Randi $\overset{´}{c}$ index, *H* index, *SDD* index, *I* index, and *AZI* index. The molecular structures of the four polysaccharides are compared graphically based on these nine degree-based topological indices. It is important to note that polysaccharides are a type of biopolymer and have diverse applications, particularly in food preservation, the pharmaceutical industry, and petroleum extraction. The findings of this research will be valuable for chemists and pharmaceutical researchers in their respective fields of study. The results of this investigation can have various applications in the field of polymer science and material engineering. By analyzing the topological indices of guar gum, researchers can gain insights into its molecular structure, connectivity, and properties. This information can be used to predict and understand the behavior of guar gum in different environments, such as its solubility, viscosity, and interactions with other molecules.

Furthermore, these results can help in the design and optimization of guar gum-based products and formulations. By correlating the topological indices with the performance of guar gum in various applications, researchers can tailor its properties to meet specific requirements in industries such as food, pharmaceuticals, cosmetics, and agriculture. Overall, these results can contribute to a better understanding of its structure–property relationships and facilitate the development of innovative products and technologies in diverse fields.

## 8 Future work

The authors will investigate the polysaccharide guar gum and its chemical variants with respect to the generalized reverse degree for future work.

## Data Availability

The original contributions presented in the study are included in the article/Supplementary Material; further inquiries can be directed to the corresponding author.
